# Atherogenic Risk, Anthropometry, Diet and Physical Activity in a Sample of Spanish Commercial Airline Pilots

**DOI:** 10.3390/ijerph19074128

**Published:** 2022-03-31

**Authors:** Ana Alaminos-Torres, Jesús Román Martínez-Álvarez, Noemi López-Ejeda, Maria Dolores Marrodán-Serrano

**Affiliations:** 1 Physical Anthropology Unit, Department of Biodiversity, Ecology and Evolution, Faculty of Biological Sciences, Complutense University of Madrid, 28040 Madrid, Spain; noemilop@ucm.es; 2 EPINUT Research Group (ref. 920325), Faculty of Medicine, Complutense University of Madrid, 28040 Madrid, Spain; jrmartin@med.ucm.es; 3 Spanish Society of Dietetics and Food Sciences, Pozuelo de Alarcón, 28224 Madrid, Spain

**Keywords:** abdominal obesity, atherogenic index of plasma, aviation pilots, body mass index, cardiovascular disease

## Abstract

Cardiovascular accidents are the most disabling event for pilots, causing complicated situations during flight and the withdrawal of license. The study aims to assess the modifiable risk factors and the atherogenic index of plasma (AIP) associated with anthropometric, physiometabolic and lifestyle profiles in a sample of Spanish aviation pilots. Data from pilots’ clinical and professional history, anthropometric and bioelectrical impedance assessments of nutritional status, and diet and physical activity questionnaires. The sample comprised 304 men pilots. Up to 53.6% showed excess weight, of which 6.4% were obese, 64.3% presented high relative adiposity and 64.6% showed abdominal obesity. Regarding the physiometabolic profile, 10.0% had hypertension, 42.6% hypercholesterolemia, 9.4% high LDL and 10.6% low HDL, 9.4% hyperglycemia and 8.1% hypertriglyceridemia. The adherence to the Mediterranean diet (MedDiet) was high in 29.7% and low in 14.7%. Most of the sample showed a good physical activity level. The AIP risk increased with higher obesity indicators and LDL cholesterol levels. There was an inverse relationship between the MedDiet adherence and vigorous physical activity and the risk of atherogenicity. Elevated rates of overweight, abdominal obesity and hypercholesterolemia were found, contributing to the atherogenic risk of plasma (AIP). This parameter was significantly associated with all anthropometric indicators and LDL cholesterol. Prevention plans on reducing excess fat and blood cholesterol levels are recommended to reduce cardiovascular risk in Spanish aviation pilots and ensure flight safety.

## 1. Introduction

Pilots are exposed to several risk factors during their careers. Their professional activity is highly demanding as they are responsible for flight safety. Moreover, their activity includes frequent time changes, which can affect sleep and circadian rhythms and have differing impacts on lifestyle habits [1,2].

Since the International Civil Aviation Organization set out the physical capacity of commercial airline pilots’ regulations, these professionals must undergo a yearly medical examination [3] to renew their licenses. One of the most concerning aspects of incapacitation, especially during the flight, is cardiovascular events [4,5]. These are also one of the leading causes of disability and loss of licenses among commercial pilots [6]. Some of the causes of sudden incapacitation are myocardial infarction, cardiac arrhythmia and seizures [7]. In addition, one study reported that more than half of the cases of disability occurred suddenly, with almost 50% suffering from premature ischemic heart disease, revealing the significance of cardiovascular events among such professionals despite the strict medical screening process [8].

Some known risk factors related to cardiovascular disease (CVD) are obesity, high blood pressure or other metabolic alterations such as insulin resistance, high triglycerides or hypercholesterolemia. At a physiological level, excessive triglyceride deposition triggers adipocyte hypertrophy causing local inflammation that activates macrophages in the adipose tissue which secrete cytokines and chemokines. This promotes the activation of monocytes in the circulatory system, generating a low-intensity systemic inflammatory reaction [9,10] and a high level of oxidative stress [11]. These alterations would contribute to the development of insulin resistance, cardiovascular risk and other obesity-related morbidities [12,13].

One biomarker strongly associated with atherosclerosis and cardiometabolic health is the atherogenicity index of plasma (AIP) [14,15]. High levels of this parameter have been related to ischemic heart disease [16] or coronary artery disease [17].

There are also other modifiable lifestyle-related factors such as diet and physical activity. Their imbalance is frequently associated with excess weight and abdominal obesity, related to cardiovascular morbidity and mortality [18,19].

The above evidence motivated the present study that aims to determine the prevalence of risk factors related to CVD and analyze the atherogenic index associated with the anthropometric and physiometabolic profile, diet quality and physical activity in a sample of Spanish aviation pilots.

## 2. Materials and Methods

### 2.1. Ethical Considerations

The project was approved by the Clinical Research Ethics Committee of the San Carlos Clinical Hospital in Madrid and followed the ethical principles set out in the World Medical Association’s Helsinki Declaration [20]. Informed consent was obtained from each participant.

### 2.2. Participants and Data Collection

A general call for participation in the study was made to the members of the Spanish Professional Association of Commercial Aviation Pilots (COPAC) and the Spanish Union of Airline pilots (SEPLA). Pilots who wanted to voluntarily participate were evaluated in the period between October 2019 and February 2020. Only pilots of commercial aircraft in active service who had passed the last medical check-up were included. A total of 304 male pilots aged between 24 and 64 years were analyzed. Women were not considered because the sample was too small. The personal interview consisted of questions related to the pilots’ professional history. Pilots were classified in two types according to the distance traveled. Long-haul pilots, those that work on transcontinental flights, and short-haul or medium/short-haul pilots.

### 2.3. Anthropometry and Bioimpedance

Height (cm), weight (kg) and waist circumference (WC)(cm) were collected according to the International Biological Program [21]. The body mass index (BMI) and the waist-to-height ratio (WHtR) were calculated. To assess the nutritional status, the BMI was classified following the World Health Organization criteria [22]. Central obesity was determined when the WHtR was ≥0.5 according to Hsieh and Muto [23]. Body fat percentage (BF%) was analyzed using electric tetrapolar bioimpedance (Tanita BC558) and classified according to Gallagher et al. [24].

### 2.4. Physiometabolic Parameters and the Atherogenic Risk

The data on blood concentration of glucose (mg/dL), total cholesterol (mg/dL), HDL cholesterol (mg/dL), LDL cholesterol (mg/dL) and triglycerides (mg/dL) were collected from the most recent clinical analysis provided by the participants. Although blood pressure could not be measured in situ, each participant was asked whether they had hypertension and/or took medication to control it. The guidelines of the International Diabetes Federation (IDF) [25] were followed to establish hyperglycemia (>100 mg/dL) and hypertriglyceridemia (≥150 mg/dL). Hypercholesterolemia (≥200 mg/dL), HDL cholesterol (abnormal level for men < 35 mg/dL) and LDL cholesterol (normal-high: >100 and <160 mg/dL and high: >160 mg/dL) was classified following the Spanish Heart Foundation criteria [26]. The AIP was calculated using the formula proposed by Frohlich and Dobiasova [27] (log10 (TG/HDL-C)). The cut-off points considered were low cardiovascular risk when AIP is ≤0.11; medium: ≥0.12 and ≤0.21 and high: >0.21 [28].

### 2.5. Lifestyle Factors

To measure adherence to a Mediterranean diet (MedDiet), we administered the 14-item PREDIMED questionnaire [29], and the total score was classified as: low adherence, 0–7 points; medium adherence, 8–10; high adherence, ≥11. The International Physical Activity Questionnaire (IPAQ) was used to gather information on physical activity [30]. This instrument allows the classification of the daily activity into three intensity categories (low, moderate, vigorous). To calculate the metabolic equivalents (MET), each type of activity was associated with a value multiplied by the days and minutes reported for that activity. Using the MET, the total physical activity was classified as low (<600 MET), moderate (600 to 3000 MET), or high (>3000 MET) [31]. The sedentary level was the time in hours spent sitting during a working day.

### 2.6. Statistical Analyses

The Kolmogorov–Smirnov test was used to examine normality. To compare means between groups, a one-way ANOVA was applied with a Bonferroni-adjusted post hoc pairwise two-sided test to avoid type I error. To compare the nonparametric variables, the Mann–Whitney U test was applied. When the differences between groups yielded differences with a *p*-value less than 0.05, they were considered statistically significant. The medians, 25th, 75th percentiles, maximum and minimum values of each continuous variable with respect to the risk levels calculated for the AIP were represented by boxplots. The chi-square test was used to contrast proportions between categorical variables. Statistical analyses were performed using SPSS v.24.

## 3. Results

### 3.1. Professional Characteristics

In total, 48.7% (*n* = 148) of the pilots performed short/medium-haul, while 51.3% (*n* = 156) performed long-haul trips. The mean age was 48.30 ± 10.25 years, being lower in short/medium-haul pilots (45.92 ± 10.31 years) than in their long-haul counterparts (50.20 ± 9.51 years) (Mann–Whitney U: 8050.00, *p* < 0.001). The mean total flight hours/year was 12,592.14 ± 6278.99. The number of nights per month spent away from their habitual residence was 9.36 ± 4.4. As expected, the pilots currently on long-haul flights had greater experience, with more total flight hours on average (14,334.64 ± 5688.44), compared to short/medium-haul pilots (10,815.93 ± 6356.99) (Mann–Whitney U: 7408.500, *p* < 0.001), and they spent more nights per month away from their habitual residence (10.12 ± 3.08 vs. 8.45 ± 5.48, Mann–Whitney U: 65,000.00, *p* < 0.001).

### 3.2. Anthropometry and Bioimpedance

More than half of the sample (53.6%) presented excess weight, 47.2% being overweight and 6.4% having obesity. The mean BMI was 25.57 ± 3.09 kg/m^2^. The mean WC was 92.19 ± 10.73, and regarding the WHtR, the sample mean was 0.521 ± 0.057. A substantial proportion of the pilots presented abdominal obesity, according to the WHtR (64.6%), and the mean BF% was 23.83 ± 6.58, with 64.3% of high relative adiposity. Table 1 shows the comparative analysis of the anthropometric profile and the relative adiposity according to flight type. There were no significant differences between short/medium and long-haul pilots on any direct or derived anthropometric measures or for the BF%.

### 3.3. Physiometabolic Parameters and Atherogenic Index of Plasma

The self-reported high blood pressure rate was 10% for the total sample, affecting 6.9% of the short/medium-haul pilots and 13.3% of the long-haul group (chi-square: 3.34; *p* < 0.05). The most prevalent altered metabolic parameter was total cholesterol, with 42.6% of pilots exceeding the upper limit of 200 mg/dL recommended by the FEC23. The prevalence of LDL cholesterol above 160 mg/dL was 9.4% and HDL cholesterol under 35 mg/dL showed a rate of 10.6%. The percentage of pilots with hyperglycemia was 9.4% and with hypertriglyceridemia, 8.1%. The mean AIP for the total sample was 0.178 ± 0.253. Regarding the prevalence of the AIP risk, it was low for 38.5%, 15.4% presented an intermediate risk and 46.2% a high risk. There were no significant differences between the AIP averages according to the type of flight. Table 2 presents the mean values for the metabolic variables showing no significant differences for any of these parameters according to the type of flight.

### 3.4. Adherence to Mediterranean Diet

The mean score on the PREDIMED questionnaire was 9.49 ± 1.94, with no difference between flight types. In our sample, 14.7% of the pilots presented low adherence, 55.6% medium adherence and 29.7% high adherence. An analysis of each item on the questionnaire revealed that the lowest scores were in item 9 (“How many servings of legumes do you consume per week?”), item 4 (“How many fruit units (including natural fruit juices) do you consume per day?”) and item 10 (“How many servings of fish or shellfish do you consume per week?”). The item 1 was the one that obtained the highest score.

### 3.5. Physical Activity and Sedentarism

The reported level of physical activity was low in 4.4% of the pilots, moderate in 38.2% and high in 57.3%. No differences were found for the MET expanded in the different types of activities between the pilots according to flight type, but we found significant differences in the hours spent sitting on a working day, which was higher in long-haul pilots with more than two hours apart on average (Table 3).

### 3.6. AIP Interaction with Age, Cardiometabolic Profile and Lifestyle Factors

**Figure 1:**
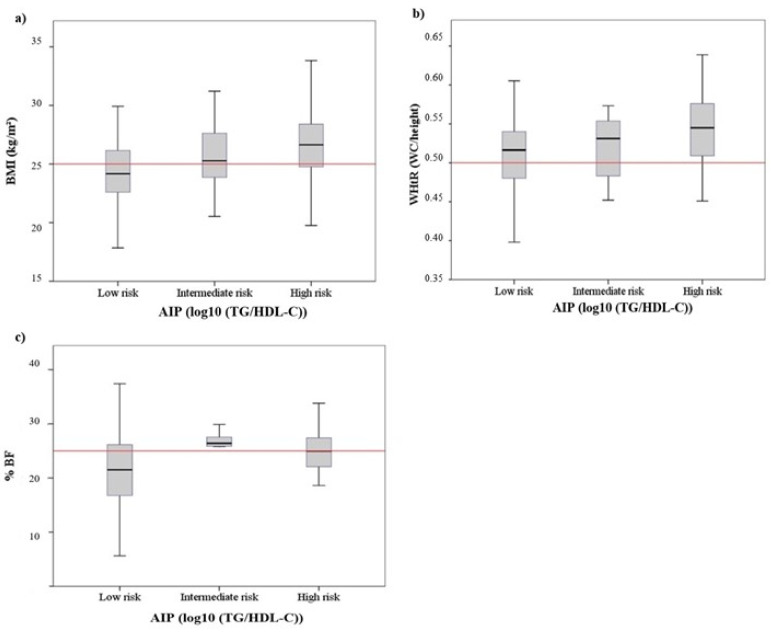
Anthropometric parameters boxplots: (**a**) BMI, (**b**) WHtR and (**c**) %BF, according to the different levels of AIP.

**Figure 2:**
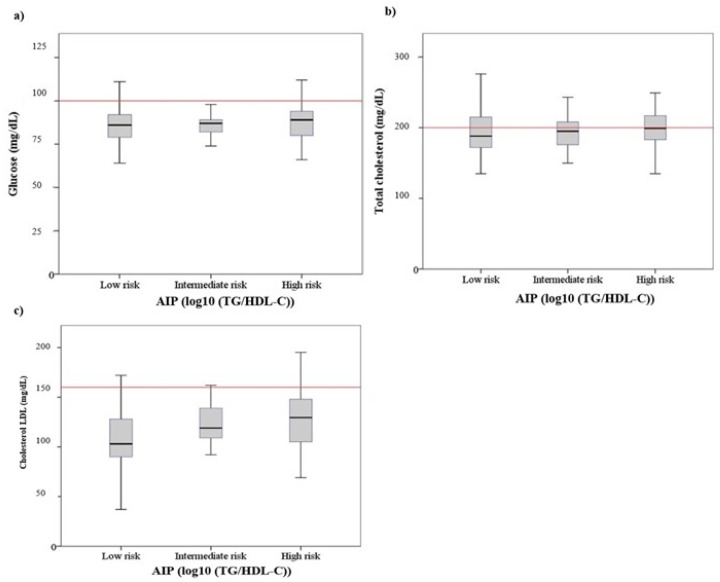
Boxplots of (**a**) glucose, (**b**) total cholesterol and (**c**) cholesterol-LDL, according to the different levels of AIP.

**Figure 3:**
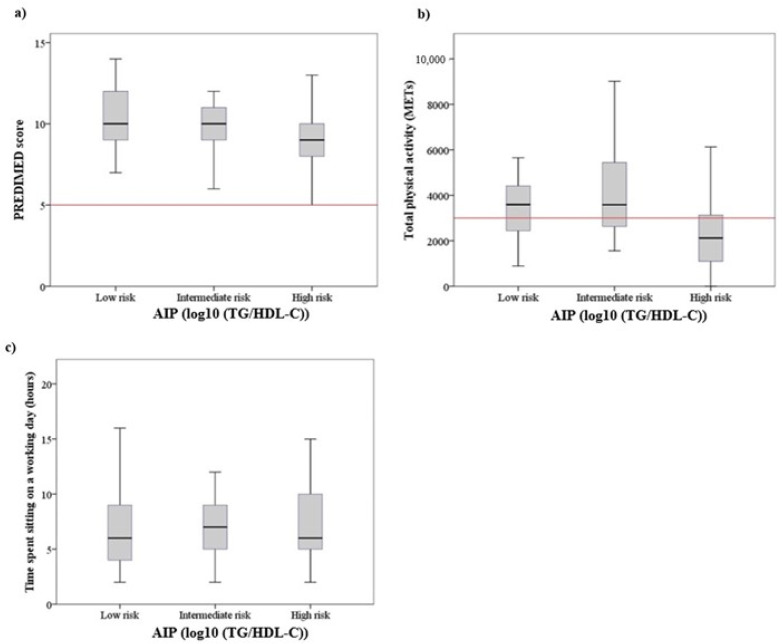
Boxplots of (**a**) adherence to MedDiet, (**b**) physical activity and (**c**) the time spent sitting in a working day, according to the different levels of AIP.

The age of pilots did not show significant differences when comparing the three atherogenic risk categories (F: 0.364, *p* = 0.695). No differences were detected between the prevalence of arterial hypertension (chi-square: 0.462, *p* = 0.794). As shown in Figure 1, the mean values for the anthropometric indicators of obesity increased as a function of the risk category established for the AIP (BMI: F: 8.35, *p* < 0.001; WHtR: F: 7.70, *p* < 0.001; %BF: F: 4.54, *p* < 0.05). In all three indicators, the Bonferroni post hoc test showed differences between low- and high-risk categories (BMI mean difference (MD): −2.23, *p* < 0.001; WHtR MD: −0.40, *p* < 0.001; %BF MD: −4.75, *p* < 0.05). Figure 2 shows the association between serum metabolic levels and risk categories for AIP. Only the LDL cholesterol levels were significantly increased between the lowest to highest atherogenic risk categories (F: 8.60, *p* < 0.001; Bonferroni test: MD: −20.11, *p* < 0.001). As shown in Figure 3, there was an inverse relationship between the MedDiet adherence score and the risk of atherogenicity (Mann–Whitney U: 1301.50; *p* < 0.05). On the other hand, the average METs expended was higher in the low-risk AIP category than the high-risk category (F: 7.302, *p* < 0.001, Bonferroni test: MD: 1620.61, *p* < 0.001). Figure 3 also shows that there was no association between the number of hours sitting and the atherogenic risk category.

## 4. Discussion

The present work analyzed various aspects of lifestyle and health in a sample of Spanish aviation pilots, a group of professionals that had been barely studied from this perspective. In this study, no significant differences were found for any anthropometric, physiometabolic, MedDiet adherence, or physical activity parameters according to the type of flight. Therefore, no distinction can be made in the cardiovascular risk profile between the two groups. Although significant differences were found in the time spent sitting, being greater among those who flew long-haul flights, no association was found with AIP.

Excess weight, particularly abdominal obesity, has been associated with some chronic diseases, of which cardiovascular disorders are predominant, as well as diabetes and certain types of cancer. Moreover, it contributes to a reduced quality of life and life expectancy [32,33]. The total excess weight in pilots presented a rate practically identical to that reported for the general population in Spain (53.9%), in a comparative study among European countries [34]. However, the prevalence of obesity reported in that study (17.1%) was three times higher than in the current study.

Compared to other professionals, the mean BMI of Spanish pilots was similar to that found in 354 pilots of Swedish airlines (BMI: 25.2 kg/m^2^). Moreover, the prevalence of overweight and obesity was comparable, 41% and 4%, respectively [35]. A similar overweight prevalence was found in a study that collected 8588 examinations for the licensing of UK pilots (46.8%), however, the obesity was twice as high (12.4%) [36]. Regarding the WHtR, the mean in this sample was more favorable than in 2913 men from the Canary Islands [37]. It is important to highlight that this index has been a useful parameter in detecting different components associated with cardiovascular risk; in fact, it has been observed that individuals with a BMI below 30 kg/m^2^ may have excess abdominal fat and therefore be candidates for cardiometabolic risk [38]. Furthermore, the visceral fat depot has a worse prognosis of cardiovascular and type-2 diabetes morbidity than general obesity [39,40] and is highly associated with atherogenicity [27,41,42,43].

One of the critical risk factors for CVD is hypertension; therefore, prevention is fundamental during the development of the pilot’s career. The prevalence of hypertension in this sample was remarkably low (10%), compared to the Spanish general population in a study where the prevalence was 26.7% in men [44]. Likewise, hypertension were almost three times higher in the study of cardiovascular risk factors in the UK (28.7%) [36]. However, an analysis of Colombian pilots reported a lower prevalence for this physiological parameter (7.8%) [45]. Dyslipidemia is characterized by alterations in the concentration of lipids/lipoproteins which are closely related to atherosclerosis development [46]. The prevalence of a high total cholesterol was lower than in the ENRICA study (50.5%) [47], but no less worrying, given that it affected 42.6% of the pilots. However, comparing it with a study conducted in 216,914 Spanish workers, all lipid parameters were lower, especially the high LDL cholesterol (9.4% vs. 19.3%) and low HDL cholesterol (10.6% vs. 24.9%), but also high total cholesterol (42.6% vs. 48.7%) and high blood triglyceride level (8.1% vs. 10.8%), showing that the cardiovascular risk was elevated generally, but mainly in certain types of employments [48]. Similarly, hyperglycemia was slightly lower here (9.4%), compared with the values reflected in a review of different studies, where the prevalence of type 2 diabetes mellitus was estimated between 10 and 15% [49]. In addition, the Di@bet.es study found that the overall prevalence of diabetes mellitus was 13.8% [50]. Likewise, the prevalence was almost twice less than that observed in 5226 Spanish adults attending dietary consultations (15%) [51]. Regarding hypertriglyceridemia, in the aforementioned study, the proportion of men with this pathology was 14.9%, almost double that observed in the present study (8.1%).

The literature has shown that a MedDiet helps avoid metabolic syndrome and prevents various cardiovascular events, decreasing mortality-related risk [52,53]. One of the critical elements of a MedDiet is extra virgin olive oil, which has anti-inflammatory properties, among others [54]. The PREDIMED studies have conclusively shown that greater adherence to a MedDiet protects against cardiovascular disease [53]. The assessment of MedDiet adherence in university students in Murcia showed a need to increase the intake of fruit, vegetables and legumes, these items scoring the lowest, as in this study [55]. In the PREDIMED study involving 7447 participants, the mean score was 8.7 ± 2.0 for men, lower than the one reported here. Moreover, they found an inverse relationship between adherence to MedDiet and obesity indices such as %BF or WHtR [29]. Regarding physical activity, both groups of pilots reported a greater total physical activity than that reported in the general population. For example, in a sample for the Di@bet.es study, sedentariness exceeded 30%. Furthermore, the total physical activity was considerably lower than in this sample [56]. In addition, another study carried out in 20 countries found that, in the Spanish sample, the rate of low-intensity physical activity was 24.2%, medium 36.2%, and high 39.6% [57], while in our sample, the rate of low-intensity physical activity was substantially lower and there was a greater prevalence of moderate-intensity and vigorous physical activity.

The association of AIP with other factors showed that the higher the BMI, WHtR or BF%, the higher risk of AIP. Similarly, a cross-sectional study with 1000 participants found that AIP was significantly correlated with WC, a higher BMI, and a lower level of physical activity, proposing the AIP as a marker of cardiovascular disease [58]. Another study analyzed the association between factors related to cardiovascular diseases, such as BMI, total cholesterol and fasting blood glucose, showing that AIP increased as these parameters increased, with the most robust correlations between AIP and BMI and total cholesterol. As expected, AIP was found to be significantly lower in the group that engaged in regular physical activity [41]. The participants with the poorest cardiovascular health status were found to have a higher risk of developing atherosclerosis [41]. Another longitudinal study determined the association of AIP with cardiometabolic disorders. Among men, they found that insulin levels, obesity and cholesterol were the main determinants, and higher quartiles of AIP significantly predicted coronary heart disease, diabetes and hypertension [59]. In another sample of 2701 adults, moderate-to-vigorous physical activity was independently related to the likelihood of having an elevated AIP, with this relationship being mediated by central adiposity [60]. In the present study, the pilots who engaged in the highest level of physical activity had a lower prevalence of elevated AIP. This relationship was not so evident in the case of moderate exercise. Nonetheless, the anthropometric parameters associated with central adiposity also suggested a lower risk of high AIP when such parameters were below the established cut-off points for the obesity condition. Regarding dietary patterns, our findings suggest that the greater the adherence to a MedDiet, the lower the risk of AIP.

The results showed that the related cardiovascular factors of pilots are comparatively better than in the general Spanish population. However, further attention and prevention should be paid to this group, especially since their work is highly demanding. Additionally, the AIP could be considered a cardiovascular risk biomarker among pilots. Likewise, we highlight the importance of promoting healthy lifestyle habits to prevent excess weight or abdominal obesity to improve cardiovascular status.

The present study is not without limitations, one of which is the small sample size, so the results cannot be extrapolated to the entire professional group. Nonetheless, we collected data on numerous variables for each participant, serving to enrich the study. As the participation was voluntary, the data may be partially biased, with individuals more concerned about their health the most willing to attend interviews. Likewise, the time elapsed between the blood test performed in a laboratory as part of the last medical check-up and the rest of the parameters analyzed in the study may vary among participants, never exceeding eleven months. In that period, changes in habits could have occurred. Drug consumption was not recorded, so there could be cases of normal biochemical profile but pharmacologically controlled. Furthermore, the precise data on blood pressure were not included in the clinical reports and therefore, its association with atherogenic risk and lifestyle factors could not be analyzed. However, the study has several strengths, such as using personal interviews, collecting anthropometric data in situ and gathering data from reliable clinical sources.

## 5. Conclusions

This study provides unprecedented data on the anthropometric, atherogenic and lifestyle profile of Spanish aviation pilots. These professionals presented lower levels of hypertension, hyperglycemia and hypertriglyceridemia, a greater adherence to a MedDiet and a higher level of physical activity than the general Spanish population. However, they presented worrying rates of overweight, abdominal obesity and hypercholesterolemia contributing to an atherogenic risk. The results showed that AIP could be an effective and simple biomarker to monitor CVD in this professional group. The results obtained may help design specific prevention plans focused on reducing excess adipose and the reduction of blood cholesterol levels, the main predisposing factors to CVD in Spanish aviation pilots. It is crucial to continue research in this direction to safeguard the health of pilots during their careers and ensure flight safety.

## Figures and Tables

**Table 1:** Anthropometric profile according to flight type.

	Short/Medium-Haul Pilots	Long-Haul Pilots	
Anthropometric Variables	Mean ± SD	Mean ± SD	*p*-Value (*t*-Test)
Height (cm)	177.03 ± 7.64	176.99 ± 6.91	0.964 ^NS^
Weight (kg)	79.92 ± 11.66	80.57 ± 11.96	0.645 ^NS^
Waist circumference (cm)	92.09 ± 10.55	92.28 ± 10.99	0.901 ^NS^
Body mass index (kg/cm^2^)	25.45 ± 3.016	25.67 ± 3.17	0.540 ^NS^
Waist-to-height ratio	0.521 ± 0.0559	0.52 ± 0.059	0.982 ^NS^
Body fat (%)	23.94 ± 4.63	23.64 ± 7.75	0.813 ^NS^

NS: nonsignificant; SD: standard deviation.

**Table 2:** Metabolic profile according to flight type.

	Short/Medium-Haul Pilots	Long-Haul Pilots	
Metabolic Variables	Mean ± SD	Mean ± SD	*p*-Value (*t*-Test)
Glucose (mg/dL)	87.77 ± 8.91	88.216 ± 13.024	0.799 ^NS^
Total cholesterol (mg/dL)	202.17 ± 36.87	195.21 ± 30.82	0.162 ^NS^
HDL cholesterol (mg/dL)	57.37 ± 15.26	60.38 ± 20.45	0.302 ^NS^
LDL cholesterol (mg/dL)	122.64 ± 28.40	118.32 ± 29.24	0.348 ^NS^
Triglycerides (mg/dL)	89.76 ± 37.25	95.24 ± 44.67	0.388 ^NS^
Atherogenic index of plasma	0.175 ± 0.245	0.181 ± 0.263	0.891 ^NS^

NS: nonsignificant; SD: standard deviation.

**Table 3:** Levels of physical activity and sedentarism according to flight type.

	Short/Medium-Haul Pilots	Long-Haul Pilots	
Activity Variables	Mean ± SD	Mean ± SD	*p*-Value (*t*-Test)
Low-intensity activity (MET)	1011.69 ± 856.96	1184.83 ± 901.66	0.141 ^NS^
Moderate-intensity activity (MET)	706.85 ± 767.26	856.47 ± 856.48	0.180 ^NS^
Vigorous activity (MET)	1858.74 ± 1728.24	2190.24 ±2152.68	0.190 ^NS^
Total activity (MET)	3628.99 ± 2476.27	4292.21 ± 3165.59	0.090 ^NS^
Time spent sitting in a working day (hours)	6.20 ± 3.97	8.84 ± 13.88	0.007 *

* Statistically significant; NS: nonsignificant; SD: standard deviation.

## Data Availability

The dataset used during the current study is available from the corresponding author on reasonable request.
